# Genome-Wide Identification of the *WRKY* Gene Family and Functional Characterization of *CpWRKY5* in *Cucurbita pepo*

**DOI:** 10.3390/ijms25084177

**Published:** 2024-04-10

**Authors:** Junhong Chen, Fei Tao, Yingyu Xue, Bingliang Xu, Xiaowei Li

**Affiliations:** 1 College of Plant Protection, Gansu Agricultural University, Lanzhou 730070, China; juniorc1110@163.com (J.C.); skyfit-1985@163.com (F.T.); lixiaow@gsau.edu.cn (X.L.); 2 Biocontrol Engineering Laboratory of Crop Diseases and Pests of Gansu Province, College of Plant Protection, Gansu Agricultural University, Lanzhou 730070, China

**Keywords:** *Cucurbita pepo*, *WRKY*, phylogenetic analysis, seed coat development

## Abstract

The *WRKY* gene family is crucial for regulating plant growth and development. However, the *WRKY* gene is rarely studied in naked kernel formation in hull-less *Cucurbita pepo* L. (HLCP), a natural mutant that lacks the seed coat. In this research, 76 *WRKY* genes were identified through bioinformatics-based methods in *C. pepo*, and their phylogenetics, conserved motifs, synteny, collinearity, and temporal expression during seed coat development were analyzed. The results showed that 76 *CpWRKYs* were identified and categorized into three main groups (I−III), with Group II further divided into five subgroups (IIa−IIe). Moreover, 31 segmental duplication events were identified in 49 *CpWRKY* genes. A synteny analysis revealed that *C. pepo* shared more collinear regions with cucumber than with melon. Furthermore, quantitative RT-PCR (qRT-PCR) results indicated the differential expression of *CpWRKYs* across different varieties, with notable variations in seed coat development between HLCP and CP being attributed to differences in *CpWRKY5* expression. To investigate this further, *CpWRKY5*-overexpression tobacco plants were generated, resulting in increased lignin content and an upregulation of related genes, as confirmed by qRT-PCR. This study offers valuable insights for future functional investigations of *CpWRKY* genes and presents novel information for understanding the regulation mechanism of lignin synthesis.

## 1. Introduction

*Cucurbita pepo* L., commonly known as zucchini, is an annual plant native to Mexico and North America [1]. It is an economically valuable member of the cucurbit fruit family. Because of its carbohydrates, non-cellulosic polysaccharides, phenol, flavonoid contents, and amino acids, as well as its high contents of vitamins (such as vitamin A, E, and C), *C. pepo* has been recognized as a functional vegetable [2]. In the 1880s, a naturally mutated, hull-less variety of *C. pepo* (HLCP) was discovered in Austria [3]. Using scanning electron microscopy, Bezold et al. reported that the formation of this HLCP variety occurs due to the absence of lignification and the collapse of the outer four cortices (namely epidermis, subcutaneous tissue, sclerenchyma, and parenchyma) compared with the hulled variety of *C. pepo* (CP) [4].

*Cucurbita pepo* seed oil and seed extract have demonstrated remarkable nutritional benefits and medicinal properties, particularly in addressing benign prostatic hyperplasia and inhibiting the proliferation of human papillary thyroid cancer cells [5,6]. CP seeds must be mechanically shelled before eating and processing at a later stage, which increases the production cost. In contrast, HLCP seeds are easy to eat and process, and complete seeds can be obtained without shelling, making them the seeds of choice by the industry [7,8]. According to studies investigating the phenotypic traits and physiological and biochemical parameters associated with HLCP and CP seed coat development, HLCP is formed because of the lack of lignin accumulation [9]. Molecular mechanism studies have shown that CP and HLCP seed coat development is controlled by a pair of nuclear genes present at the same locus. However, some studies have inferred that HLCP seed coat development is controlled by a pair of major genes and some modified genes that control particular quantitative traits [3,10,11,12]. Accordingly, researchers believe that a single gene is involved in seed coat lignification, and the phenotype of HLCP mutants resulted from the combined action of lignin-synthesis-related gene expression and many environmental factors [13,14].

As major modulators of gene expression, transcription factors (TFs) are essential for the development of plant tissues and the signal transmission [15,16]. The *WRKY* TF family is one of the largest and most well-researched in higher plants [17]. Following the discovery of the first *WRKY* gene in sweet potato (*Ipomoea batatas*: SPF1), *WRKY* genes have since been found in numerous plant species and extensively studied [18], including *Dendrobium catenatum* (62 *DcWRKYs*) [19], *Cucumis sativus* L. (61 *CsWRKYs*) [17], *Citrullus lanatus* (63 *ClWRKYs*) [20], and *Taraxacum kok-saghyz* Rodin (72 *TkWRKYs*) [21].

The WRKY DNA-binding domain (DBD) is defined by the N-terminus invariant heptad WRKYGQK amino acid motif and the C-terminus C_2_H_2_ or C_2_HC zinc-binding motif. When *WRKY* TFs bind to the (T)TGAC(C/T), that is, the W-box cis-element in the target gene promoter, the expression of *WRKY* genes is induced to achieve cellular homeostasis [22]. WRKY TFs can be categorized into three groups based on the number of WRKY domains and the type of zinc finger motif they possess. Group I WRKY proteins typically contain two WRKY domains, whereas Group II WRKY proteins possess a single WRKY domain and are further classified into five subgroups (IIa–IIe). Both Group I and Group II proteins feature a C_2_H_2_ zinc finger motif (C-X_4-5_-C-X_22-23_-H-X_1_-H). Group III proteins have one WRKY domain, followed by a C2HC zinc finger motif (C-X_7_-C-X_23_-H-X_1_-C) [23,24].

*WRKY* TFs play an important role in regulating different aspects of seed coat formation, including hemicellulose, cellulose, and lignin contents [25], as well as secondary cell wall deposition [26,27]. Recent research using an RNA-Seq method by Xue et al. revealed that *WRKY* TFs are also involved in the development of seed coats in *C. pepo* [28]. Among the identified *WRKY* TFs, seven were significantly upregulated in CP and two were significantly upregulated in HLCP. However, according to the study of Xue et al., *WRKY* is speculated to play an important role in the seed coat formation [28]. However, The identification of the *WRKY* gene and its function and mechanism in the development of *C. pepo* seed coat have not been studied. Therefore, we performed a genome-wide identification of *WRKYs* in *C. pepo*, and comprehensively studied their classification, conserved protein domains, chromosomal location, phylogeny, motif composition, structure, and duplication events. Moreover, we investigated the expression profile of *WRKY* genes between CP and HLCP and explored their potential functions during lignin synthesis and accumulation. These results provide the potential candidate genes for further functional analyses and enrich our understanding of the molecular mechanism underlying lignin synthesis in plants.

## 2. Results

### 2.1. Identification and Evaluation of Chromosomal Location, Multiple Sequence Alignment, and Phylogenetic Relationships of CpWRKY Proteins

**Figure 1:**
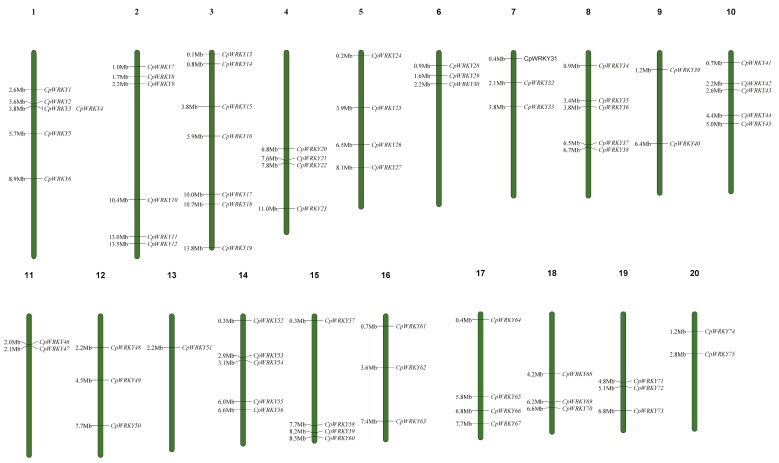
Distribution of *CpWRKY* genes on chromosomes. At the top of every chromosome is the number of that chromosome. On the right side of every chromosome are the names of the genes. The distance in mega bases (Mb) between genes or from the gene to the end of the chromosome on the left side of each chromosome.

Table S1 presents a total of 76 putative *CpWRKY* genes. Among these genes, all except *CpWRKY76* could be mapped to chromosomes 1–20. These genes were renamed as *CpWRKY1*–*CpWRKY75* according to their order on the chromosomes. (Figure 1). *CpWRKY76* could not be definitively assigned to any chromosome in the zucchini genome. Chromosome 3 harbored the highest number of *WRKY* genes (7), whereas chromosome 13 had the lowest number of *CpWRKY* genes (1). In addition, six chromosomes (6, 7, 12, 16, 18, and 19) had three *CpWRKY* genes, while chromosomes 1 and 2 harbored relatively more genes than chromosomes 9, 11, and 20. This indicated that, despite the presence of *CpWRKYs* on all 20 chromosomes, their distribution across respective chromosomes was uneven.

**Figure 2:**
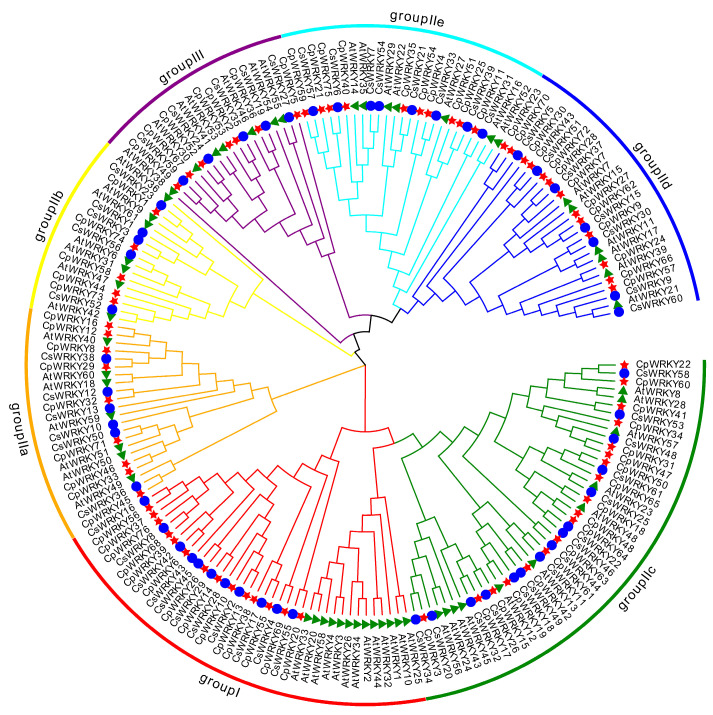
Phylogenetic analysis and family classification of WRKY domains. Distinct arcs of various colors show diverse groups of the WRKY domain. The WRKY domains from *C. pepo*, cucumber, and *Arabidopsis* are denoted by a red star, blue circle, and green triangle, respectively.

To investigate the evolutionary relationship among *WRKY* genes in *C. pepo*, a phylogenetic tree was constructed based on the published WRKY proteins in cucumber and *Arabidopsis thaliana* following multiple sequence alignment [17]. The phylogenetic tree categorizes these proteins into three groups (Groups I, II, and III) based on the characteristics of the conserved domains (Figure 2). Group I included fourteen members, which contained two conserved WRKY domains, whereas CpWRKY55 protein had lost its C-terminal WRKYGQK-like stretch. Group II included forty-two members, which were further subdivided into five subgroups: IIa (*n* = 9), IIb (*n* = 5), IIc (*n* = 18), IId (*n* = 13), and IIe (*n* = 10). In this group, all members possessed a complete WRKYGQK domain except CpWRKY33 and CpWRKY71 protein, which contained a WRKYGKK domain. Moreover, Group III included seven *WRKYs* that harbored the WRKY domain and the C_2_HC-type zinc finger (Figure S1).

### 2.2. Gene Structure and Conserved Motif Analysis of CpWRKYs

**Figure 3:**
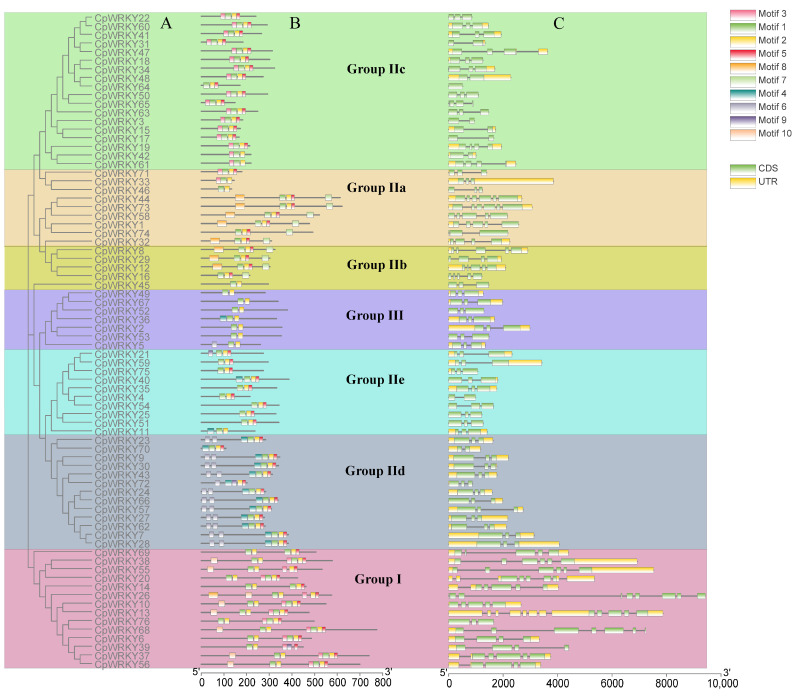
Phylogenetic grouping, conserved protein patterns, and architecture of *CpWRKY* genes. (**A**): The sequence of WRKY domain from CpWRKY protein was used to create a phylogenetic tree. Various categories and subcategories are visualized in distinct hues. (**B**): Various shaded areas indicate the pattern. (**C**): Organization of *CpWRKY* genes. The untranslated 5′- and 3′-segments, exons, and introns are depicted by a green block, yellow block, and black line, respectively.

To deepen the comprehension of *CpWRKY* gene structure through classification, an analysis of exon–intron structures and conserved motifs within these genes was conducted, followed by a phylogenetic clustering (Figure 3A). These classification outcomes aligned with those described in Section 2.1, showing that members within the same subgroup shared comparable gene structure characteristics. A total of 10 conserved motifs were identified and named as motifs 1–10. Details of the 10 putative motifs are presented in Figure S2 (Table S2). Motif 1 was found in most *CpWRKY* genes. Motifs 1 and 9 contained a WRKYGQK or WRKYGKK sequence. Group III only consisted of motifs 1, 2, 4, and 6. Most CpWRKY proteins within the same group or subgroup possessed similar motifs, and their coding sequences (CDSs) have similar numbers of introns (Figure 3B). The gene structure analysis revealed that each *CpWRKY* consisted of exons separated by variable numbers of introns. *CpWRKY* exon–intron structures were analyzed to obtain additional clues about the evolution of *CpWRKY* family members and their specific features. The introns in *CpWRKY* genes were variable in size and their number ranged from 0 to 11. Among the 76 *CpWRKY* genes identified, 8 had 8 exons, 43 had 3 exons, 7 had 4 exons, 11 had 5 exons, and 4 had 6 exons; in addition, *CpWRKY64* had 1 exon, *CpWRKY68* had 7 exons, and *CpWRKY26* had 9 exons (Figure 3C).

In terms of the number and position, the distribution patterns of exons and introns were similar within the same group. However, differences in the number of exons were observed within groups. For example, all genes in Groups IId and III had three exons, whereas most members of subfamily IIe exhibited three exons. However, *CpWRKY4* in Group IIe had two exons. Additionally, the number of exons in Group I ranged from four to nine. These findings suggest significant structural variations among *CpWRKYs*, which may correspond to the functional diversification among closely related members.

### 2.3. Synteny and Collinearity Analyses

**Figure 4:**
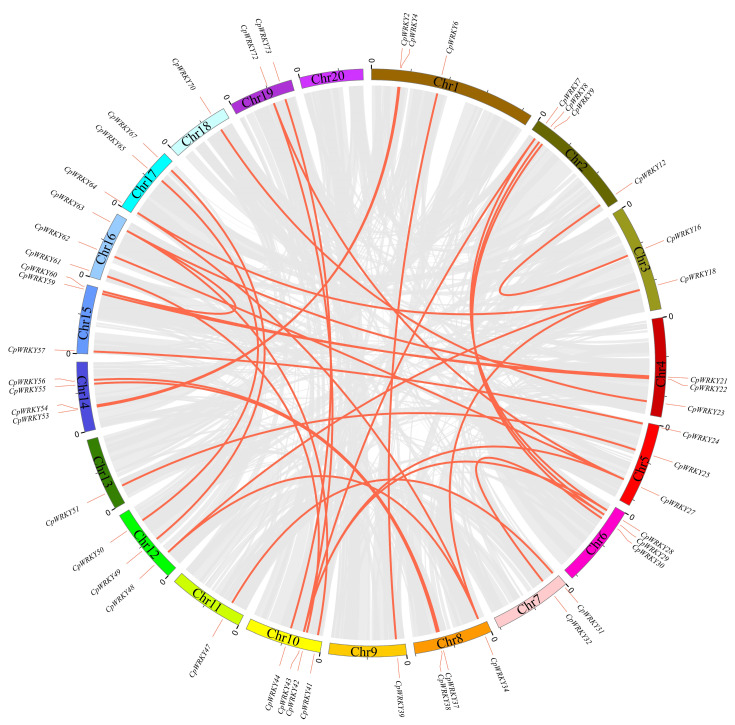
Chromosomal distribution and synteny blocks of *CpWRKY* genes. Schematic representation of the chromosomal distribution and interchromosomal relationships of *CpWRKY* genes. Gray lines indicate all synteny blocks in the *C. pepo* genome, and red lines indicate segmental duplicated *WRKY* gene pairs.

Investigating the segmental duplication events within the *WRKY* family of *C. pepo* involved an analysis of the synteny of *CpWRKY* genes using BLASTP and MCScanX. The results of the synteny analysis revealed an intricate colinear relationship among the members of the *WRKY* gene family in *C. pepo*. (Figure 4), suggesting that polyploidization is the main source of *WRKY* gene family expansion. *WRKY* members were not only identified on chromosome 20 (Table S3). Thirty-one segmental duplication events involving 49 *WRKY* genes were observed; however, no tandem duplication events were identified for these genes.

**Figure 5:**
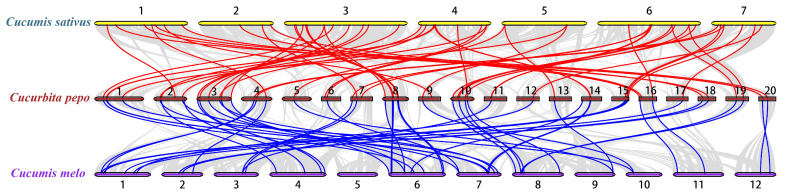
Collinearity analysis of *WRKY* gene families was conducted between *Cucurbita pepo* and representative species. The red line represents the collinearity of the *WRKY* gene family between *C. pepo* and *Cucumis sativus*, while the blue line indicates the collinearity between *C. pepo* and *Cucumis melo*. Chromosome numbers are denoted by the numbers on the lines, with other collinearities shown by gray lines.

To explore the phylogenetic mechanism of the *CpWRKY* family, we constructed a homologous map of *C. pepo* using cucumbers and melons, which belong to the same cucurbit family (Figure 5, Table S4). In total, 54 *WRKY* collinear gene pairs were identified in *C. pepo* and cucumber, followed by 49 pairs in *C. pepo* and melon, indicating that the evolutionary relationship between *C. pepo* and cucumber is more conservative than that between *C. pepo* and melon.

### 2.4. Analysis of Cis-Acting Elements in the Promoters of CpWRKYs

**Figure 6:**
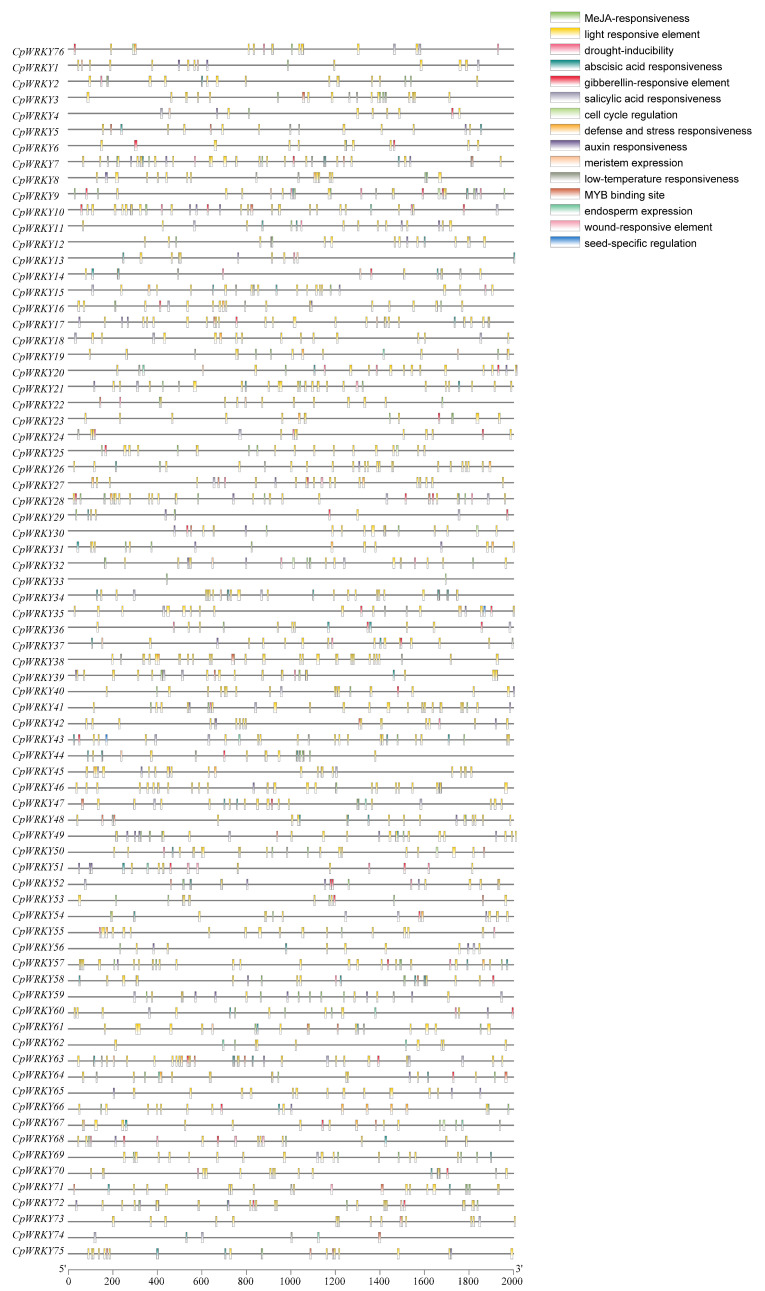
An analysis of cis-acting elements in *CpWRKY* promoters was conducted. Diverse colored boxes symbolize various cis-regulatory elements.

The *cis*-acting elements present in the promoters of *CpWRKYs* were identified and analyzed using PlantCARE. These elements, as shown in Figure 6, primarily pertain to stress responses (such as drought, wound, and low-temperature responsiveness), hormone responses (including abscisic acid, methyl jasmonate, and gibberellin responsiveness), growth and development processes (such as meristem expression, cell cycle regulation, and endosperm expression), and binding sites. Among these, the most frequently observed *cis*-acting elements were those related to light responsiveness and abscisic acid responsiveness, with 1124 and 233 elements, respectively. Moreover, a significant proportion of these elements were associated with growth and development, indicating the potential pivotal role of *CpWRKYs* in plant growth and development, as these elements were widespread and abundantly distributed in the promoters of nearly all *CpWRKYs*.

### 2.5. Expression Analysis of WRKY TFs of Seed Coat Development in C. pepo

**Figure 7:**
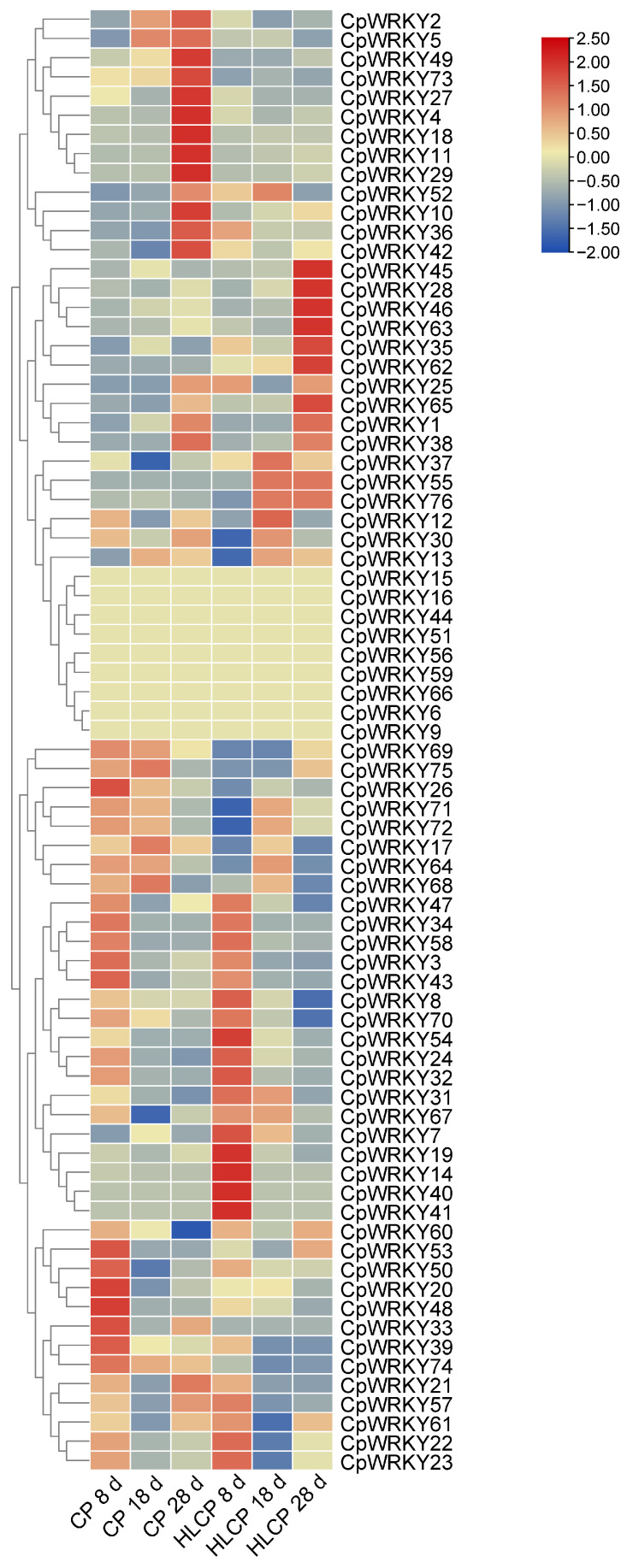
Cluster analysis of the *WRKY* gene expression profiles during seed coat development after pollination at different time points in *C. pepo*. CP: Hulled *Cucurbita pepo*; HLCP: hull-less *Cucurbita pepo*.

**Figure 8:**
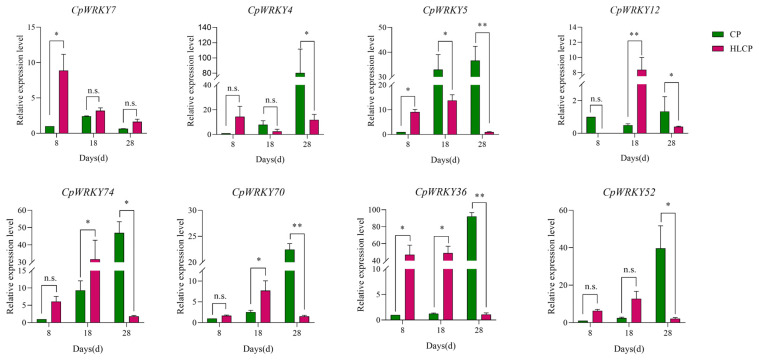
Expression pattern of *CpWRKY* genes during *C. pepo* seed coat development. CP: Hulled *C. pepo*; HLCP: hull-less *C. pepo*. n.s. represents *p*-value > 0.05. * represents significant (*p*-value < 0.05); ** represents highly significant difference (*p*-value < 0.01).

The published RNA-seq data set was used to analyze the relative expression levels of related genes in *CpWRKYs* during various seed coat development stages of *C.pepo* [28]. The analysis identified nine genes that were unexpressed in CP and HLCP seed coats at 8d, 18d, and 28d post-pollination. Among these genes, *CpWRKY56* and *CpWRKY6* were categorized in GroupI, while the remaining seven genes were placed in GroupII (*CpWRKY15*, *CpWRKY16*, *CpWRKY44*, *CpWRKY51*, *CpWRKY59*, *CpWRKY66*, and *CpWRKY9*). The expression of *CpWRKY2* and *CpWRKY5* genes showed significant differences in up-regulation between 18d and 28d post-pollination in CP. Furthermore, three genes exhibited significant variation in expression between 18d and 28d post-pollination in HLCP. There was a distinct contrast in the expression levels of 25 *CpWRKY* genes in CP after 28 days post-pollination, whereas 16 *CpWRKY* genes showed varied expression in HLCP (Figure 7 and Table S5). From Table S5 FPKM values, eight highly expressed genes were selected for qRT-PCR analysis. The expression of these genes varied between CP and HLCP across different seed coat development stages. Specifically, 8 days post-pollination, seven genes showed strong expression in HLCP: *CpWRKY4*, *CpWRKY5*, *CpWRKY7*, *CpWRKY36*, *CpWRKY52*, *CpWRKY70*, and *CpWRKY74*. Notably, *CpWRKY7* and *CpWRKY36* exhibited significantly higher expression in HLCP than in CP (*p* < 0.05). *CpWRKY4* and *CpWRKY5* displayed high expression in CP after 18 days post-pollination, with *CpWRKY5* showing notably higher expression in CP compared to HLCP. By 28 days post-pollination, the expression levels of *CpWRKY5* were significantly increased in CP compared to HLCP (*p* < 0.01; Figure 8). However, since seed coat lignification primarily occurs between 18 and 28 days after pollination, it is hypothesized that the gene *CpWRKY5* plays a crucial role in lignin synthesis in the seed coat [28].

**Figure 9:**
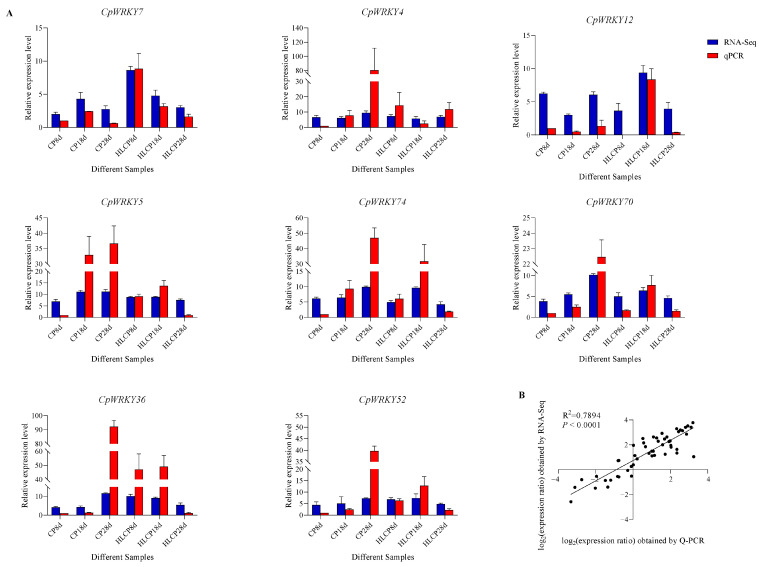
Comparison of RNA-Seq expression levels and qRT-PCR expression levels. (**A**) The expression levels of eight *CpWRKY* genes were measured using qRT-PCR and RNA-Seq FPKM analysis. The blue bar represents the relative expression level of RNA-Seq, shown as the log_2_ FPKM value. The relative gene expression of genes analyzed using qRT-PCR is represented by means of an orange histogram. (**B**) Correlation analysis of log_2_ value between RNA-Seq and qRT-PCR values.

To verify the consistency between RNA-Seq data and RT-qPCR results, log_2_FPKM was used to represent the expression level of genes in RNA-Seq. Figure 9 illustrates that the relative expression levels obtained through RNA-Seq analysis align with those obtained through qRT-PCR analysis (Figure 9A), with a correlation coefficient of 0.7894 (*p* < 0.0001; Figure 9B).

### 2.6. CpWRKY5 Positively Regulates Lignin Synthesis in Transgenic Tobacco

**Figure 10:**
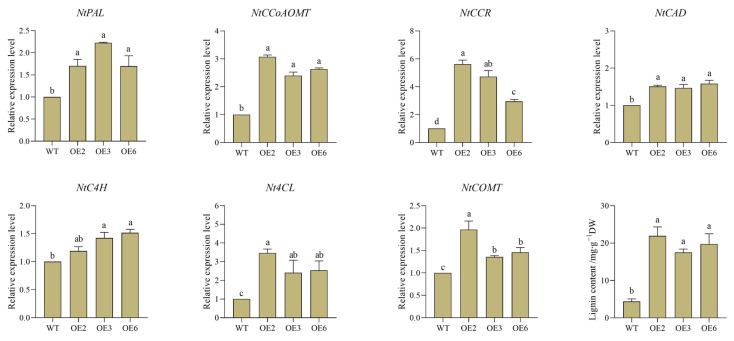
*CpWRKY5* overexpression in transgenic tobacco enhanced the lignin content and the expression of related genes. Lowercase letters indicate significant differences among different strains (*p*-value < 0.05).

The expression level of the *CpWRKY5* gene was higher in CP, with a consistent coding sequence region in both CP and HLCP (Figure S3). To clone the 786 bp *CpWRKY5* gene’s coding sequence region, cDNA from CP was used as a template, resulting in the successful construction and transformation of the pCAMBIA2300-CpWRKY5-GFP overexpression vector into tobacco (Figure S4). Three representative overexpression lines (CpWRKY5-OE2, CpWRKY5-OE3, and CpWRKY5-OE6) were selected for experiments to determine the lignin content and the expression of related genes (Figure 10, Table S6). Phenylalanine ammonialyase (*PAL*), cinnamate-4-hydroxylase (*C4H*), and 4-coumarate-CoA ligase (*4CL*) were closely related to the synthesis of total lignin content. The RT-qPCR results showed that the expression levels of *PAL*, *C4H*, and *4CL* were 1.2~3.5 times higher in the transgenic lines OE2, OE3, and OE6 compared to the WT (*p* < 0.05). Cinnamyl alcohol dehydrogenase (*CAD*) and caffeoyl-CoA O-methyltransferase (*CCoA-OMT*) are crucial catalytic enzymes in the final stages of lignin biosynthesis. The expression levels of these enzymes were observed to notably increase by 15~30% in the transgenic lines (*p* < 0.05). Furthermore, lignin content in transgenic lines was measured, and it was discovered that, following *CpWRKY5* overexpression, lignin concentration in overexpressing tobacco lines was considerably higher than that in WT (*p* < 0.05). These results indicated that *CpWRKY5* overexpression enhanced the expression of lignin synthesis genes and the accumulation of lignin content in tobacco, and *CpWRKY5* served as a positive regulator of lignin synthesis in the plant.

## 3. Discussion

*WRKY* TFs represent one of the largest TF families in plants, with multifaceted roles in plant growth and development [18]. The advancement of transgenic technology and genomics [22] has enabled the identification of the *WRKY* TF family in soybean [29], maize [30], cotton [31], cucumber [17], watermelon [20], and other species, which is of considerable significance when explaining the action mechanism of *WRKY* TFs in plants. Based on the characteristics of *WRKY* conserved motifs and zinc finger structures, Eulgem et al. classified *WRKY* genes in *A. thaliana* into groups I, II, and III. Using the same classification model [18], *Chen* et al. performed a cluster analysis of the cucumber *WRKY* gene family. In the present study, 76 *CpWRKY* family members were identified based on the *C. pepo* genome sequences [17]. The number of *WRKY* genes in *C. pepo* is higher than those in the same cucurbit plants, such as cucumber, watermelon, and melon [32,33].

Rinerson et al. proposed four major *WRKY* TF lineages in flowering plants, namely Group I + IIc, Group IIa + IIb, Group IId + IIe, and Group III, which accurately reflect the *WRKY* family evolution [34]. Based on the characteristics of the *WRKY* conserved domain and zinc finger structure, along with the phylogenetic relationship of *WRKYs* with *Arabidopsis* and cucumber genes, as well as the gene structure, amino acid sequence, and conserved domain, a total of 76 CpWRKY proteins were found to exhibit similarities with typical *WRKY* family proteins in other species. Within the *CpWRKY* family, only members of Groups IIe and IId (or Groups I and IIc) in the *CpWRKY* family were divided into two sub-branches, each involving the same branch. Group IIb, Group III, and Group IId + IIe were merged into the same clade. Group IIa is an independent branch and is believed to have directly evolved from a single-domain algae *WRKY* gene, independent of other Group I-derived lineages [34].

Domain loss appears to be common in monocots such as rice and maize, and the loss of the WRKY domain is among the divergent forces of *WRKY* gene family expansion [35,36,37]. In *C. pepo*, the C-terminal of Group I *CpWKRY55* lacked a conserved heptapeptide sequence, and the *CpWRKY39* C-terminal demonstrated the loss of the zinc finger structure. The heptapeptide motif WRKYGQK (or WRKYGKK) and the zinc finger motif are believed to be necessary for the high-affinity binding of *WRKY* TFs with the homologous cis-acting W-box element (TTGACC/T) [18]. Therefore, the heptapeptide motif mutation and the loss of the zinc finger motif may affect the normal *CpWRKY*–target gene interaction. The binding characteristics and functions of these two CpWRKY proteins need to be further explored. Whole-genome duplications, segmental duplications, and tandem duplications are crucial for gene family expansion [30]. Among 49 *CpWRKY* genes, 31 segmental duplication events were observed, but no tandem duplication events were noted. Therefore, the main driving force for *WRKY* gene expansion in *C. pepo* may be fragment replication.

Lignin, the major component of plant cell walls, is regarded as a crucial factor affecting seed coat formation. Its formation and accumulation are regulated by *WRKY* genes [38,39]. The *WRKY* family is also involved in seed coat development and lignin synthesis in *Arabidopsis* and pomegranate [40,41]. Based on transcriptome sequencing, Xue et al. reported that *WRKY* TFs were involved in seed coat formation in *C. pepo* [28]. Liping Zhang et al. found that transgenic Arabidopsis plants that overexpress *CcWRKY25* have an increased lignin content, resulting in larger plants with stronger stems [42]. It is important to note that lignin synthesis is a complex process, influenced by multiple genes and transcription factors [43]. Feng Wen et al. found that the heterologous overexpression of *Akebia trifoliata WRKY12* in tobacco resulted in suppressed lignin synthesis in key enzyme genes [44]. *CpWRKY5* overexpression resulted in the simultaneous upregulation of multiple genes encoding lignin synthesis, leading to increased lignin accumulation. Our preliminary findings suggest that *CpWRKY5* may play a role in regulating lignin synthesis; however, further investigation is required to elucidate the molecular mechanisms involved.

## 4. Materials and Methods

### 4.1. Plant Materials

Two seed varieties, namely 04LAg-26-2 (hulled *C. Pepo*, CP) and 04LAg-26-28 (hull-less *C. Pepo*, HLCP), were provided by Wuwei Golden Apple Co., Ltd. (Wuwei, China), After sowing 100% of the germinated CP and HLCP seeds in nutrient soil and vermiculite in a 2:1 volume ratio, the pots were placed in a growth chamber with a 12 h photoperiod, relative humidity of 50%, and a temperature of 25 °C [45]. Following strict self-pollination, CP and HLCP fruits were collected at 8, 18, and 28 days after pollination. The seeds of these fruits were dissected with a blade on an aseptic operation table. Finally, the seed coats were collected and frozen in liquid nitrogen and refrigerated at −80 °C [28].

The SR tobacco seeds were rinsed with sterile water, disinfected with 75% ethanol and sodium hypochlorite solution for 1 and 15 min, respectively, rinsed with sterile water 3–5 times, and dried with sterile filter paper. The dried seeds were inoculated on MS medium (pH 5.8). After 4 weeks, sterile tender leaves of the plant were collected. Their edges and main veins were removed and cut into 0.5 × 0.5 cm small pieces, which were then used as explants.

### 4.2. Identification and Characterization of Putative WRKY Genes in C. pepo

Genome and annotation files of *C. pepo* were downloaded from The CuGenDB (http://cucurbitgenomics.org/ftp/genome/Cucurbita_pepo, accessed on 20 June 2022) [46]. The hidden Markov model files (PF03106) of *WRKY* TFs downloaded from the pfam database (http://pfam.xfam.org/, accessed on 21 June 2022) were used to search all *WRKY* TFs. The hmmer3.0 software was used to search for *C. pepo* protein sequences (E ≤ 1.2 × 10^−28^), and the obtained results were deduplicated using SMART (http://smart.embl-heidelberg.de/, accessed on 21 June 2022) and NCBI-CDD (https://www.ncbi.nlm.nih.gov/Structure/cdd/wrpsb.cgi/, accessed on 22 June 2022) databases for further identification and screening. Finally, the *CpWRKY* family protein sequences were obtained [19]. The basic physicochemical properties of the obtained CpWRKY protein sequences were analyzed using ProtParam in the online tool Ex PASy (https://www.expasy.org/, accessed on 27 June 2022). The subcellular localization of these proteins was predicted and analyzed using the online software Plant-mPLoc 2.0 (http://www.csbio.sjtu.edu.cn/bioinf/plant/, accessed on 27 June 2022).

### 4.3. Sequence Alignment, Phylogenetic Tree, Classification, Gene Structure, and Conserved Motif Analyse

The *CpWRKY* gene family protein sequences obtained from *C. pepo* were aligned using ClustalW in software MEGA 7.0 and visually edited using DNAMAN to analyze the conserved WRKY core domain (60 amino acids). Using the neighbor-joining (NJ) method with 1000 bootstrap replications in MEGA 7.0 software, a phylogenetic tree of *WRKY* gene family members from *C. pepo, Arabidopsis thaliana* (*At*), and *Cucumis sativus* (*Cs*) was constructed [17].

The exon–intron structure of the *CpWRKY* gene family was analyzed using the Gene Structure Display Server (GSDS 2.0) (http://gsds.gao-lab.org/index.php/, accessed on 28 June 2022). The conserved motifs of *CpWRKY* gene family protein sequences were analyzed using the MEME (https://meme-suite.org/meme/, accessed on 28 June 2022) online tool. The parameter settings were as follows: number of motifs: 10; motif minimum width: 6; and motif maximum width: 100. Finally, the phylogenetic tree, gene structure, and conserved sequence were merged and visualized using TBtools v2.07 software.

### 4.4. Chromosomal Location, Gene Duplication, Collinearity Analyses, and Cis-Acting Elements in the Promoters of the CpWRKY Family Genes

Information about the specific location of 76 *CpWRKYs* on *C. pepo* chromosomes was obtained from the *C. pepo* genome database and visualized using Map Chart v1.0 software. Gene duplication events were analyzed using MCScanX v1.1 software. The diagram clarifying the collinearity of *CpWRKYs* with cucumber *WRKY* (*CsWRKYs*) and melon *WRKY* (*CmWRKYs*) was constructed using Circos tool [17,32]. The 2000 bp gene sequence upstream of the initiation codon (ATG) of *CpWRKYs* was identified as the gene promoter sequence in the *C. pepo* genome. *Cis*-acting elements in the promoter region were analyzed using the online PlantCARE tool and then visualized with TBtools [47].

### 4.5. Gene Expression Analysis

The Illumina RNA-seq data set (SRR15439210 to SRR15439227) was acquired from NCBI to investigate the expression patterns of *CpWRKY* genes in CP and HLCP at 8, 18, and 28 days post-pollination. Following data normalization using logarithm 2 (TPM + 1), TBtools software was utilized to generate a heatmap.

To examine changes in *CpWRKY* expression during seed development, RNA was extracted from the seed coat by using the Plant RNA Kit (Omega Bio-Tek, Guangzhou, China) according to the manufacturer’s instructions. RNA integrity and concentration were detected through 1% agarose gel electrophoresis and by using a microspectrophotometer, respectively. The first-strand cDNA was synthesized using the Revert Aid First-Strand cDNA Synthesis Kit (Thermo Fisher Scientific, Beijing, China). Specific primers for the selected eight *CpWRKY* genes were designed using Premier 5 software (Table S7). qRT-PCR was performed using the SYBR Green Premix Pro Taq HS qPCR Kit (Accurate Biology, Changsha, China) in a QuanStudio 5 Real-Time PCR System (Thermo Fisher Scientific, Beijing, China). *CpAct* was used as an internal control gene [48]. For each reaction, three independent biological and technical replicates were used. The relative expression level of each gene was calculated using the 2^−ΔΔCt^ method [49]. SPSS 21 software was used for statistical analysis.

### 4.6. Construction of Transgenic Nicotiana tabacum Overexpressing CpWRKY5

The recombinant *CpWRKY5* overexpression vector was generated by cutting the pCAMBIA2300-GFP vector with the restriction endonuclease *Sac I*/*Xba I*. By using CP intermediate cDNA as a template, the full-length CDS sequence of *CpWRKY5* was amplified through PCR. The sequence was recovered and cloned into the 35S-promoter-driven pCAMBIA2300-GFP vector. The recombinant plasmids were introduced into the *Agrobacterium tumefaciens* strain GV3101 using the heat shock method. To induce bud formation, the generated explants were co-cultured with the plasmid-containing *A. tumefaciens* GV3101 in MS + 6-BA medium for 3 days and transferred into the bud induction and differentiation medium (MS + 6-BA 1 mg/L + timentin 300 mg/L + Kan 100 mg/L, pH 5.8). When the resistant buds grew to 2 cm, they were transferred into the rooting medium (1/2MS + timentin 300 mg/L + NAA 0.1 mg/L + Kan 100 mg/L, pH 5.8) to induce root formation. After the roots were formed, the plants were transferred to pots containing nutrient soil and vermiculite (1:1) and grown at 25 °C with high humidity (>50%) under a 16 h light/8 h dark photoperiod. After the T_1_ generation grew out of the leaves, genomic DNA was extracted from the collected seeds using the TransDirect Plant Tissue PCR Kit (TransGen Biotech, Beijing, China). The positive plants and the gene expression were detected through PCR and qRT-PCR, respectively. *NtHSC70-1* was used as an internal control (Table S7) [50].

### 4.7. Determination of Lignin Content and Related Gene Expression

Primers of lignin-biosynthesis-related genes expressed in tobacco leaves during maturation (Table S7), as specified by Song et al., were used [51]. Lignin content was measured following the protocol outlined by Bruce, using a Solarbio lignin content detection kit (Solarbio, Beijing, China), with some adaptations [52]. To determine the expression of lignin synthesis-related genes in wild-type and transgenic tobacco leaves grown for approximately 4 weeks, qRT-PCR was performed using the SYBR Green Premix Pro Taq HS qPCR Kit (Precision Biology, Changsha, China) in the Quan Studio 5 Real-Time PCR System (Thermo Fisher Scientific, Inc., USA). The gene *NtHSC70-1* was used as an internal reference [50]. The experiments were carried out in triplicate, and the mean standard deviation was calculated. The relative expression level of each gene was calculated using the 2^−ΔΔCt^ method [49]. Statistical analysis was conducted using SPSS 21 software, utilizing a one-way ANOVA followed by a Tukey–Kramer post hoc test (*p* ≤ 0.05) to determine statistically significant differences. GraphPad Prism 9.0 software was employed for generating graphs.

## 5. Conclusions

In this study, 76 *CpWRKYs* were isolated and identified from the whole *C. pepo* genome. Subsequently, we systematically and comprehensively analyzed *CpWRKYs*, including their structure, phylogeny, conserved domains, chromosomal location, and duplication events, through bioinformatics analyses. The functions of the selected eight *CpWRKYs* were verified based on the transcriptome data. The construction of *CpWRKY5*-overexpressing tobacco verified the role of *CpWRKY5* as a positive regulator of lignin content and the expression of related genes. These results add to our knowledge of the *CpWRKY* gene family structure and the gene’s evolution in *C. pepo*, providing valuable insights into the mechanism underlying lignin synthesis in the seed coat in *C. pepo*. To build a framework for the molecular process of lignin synthesis, we will subsequently overexpress the candidate *CpWRKY5* in *C. pepo* and methodically investigate the regulation mechanism of *CpWRKY5* on lignin.

## Data Availability

All data supporting the findings of this study are available within the paper and within its Supplementary Materials, published online. All genome sequences and genome annotation files of *Cucurbita pepo*, *Cucumis sativus*, and *Cucumis melo* were obtained from The CuGenDB (http://cucurbitgenomics.org/ftp/genome/Cucurbita_pepo, accessed on 20 June 2022), and the published WRKY sequences of *A. thaliana* were acquired from the e TAIR database (http://www.arabidopsis.org/, accessed on 20 June 2022). The *Cucurbita pepo* seed coat transcriptome sequencing data in this study were obtained from a previous report (https://www.ncbi.nlm.nih.gov/sra/?term=SRR15439210/, accessed on 20 June 2022).

## Supplementary Materials

The following supporting information can be downloaded at: https://www.mdpi.com/article/10.3390/ijms25084177/s1.
