# Supplementary material for: Genome-Wide Identification of the WRKY Gene Family and Functional Characterization of CpWRKY5 in Cucurbita pepo
Source: Int J Mol Sci. 2024 Apr 10;25(8):4177. doi: 10.3390/ijms25084177 (PMC11049939; doi:10.3390/ijms25084177)
Supplement: Supplementary file 1 [file ijms-25-04177-s001.zip › Figure S1.pdf]

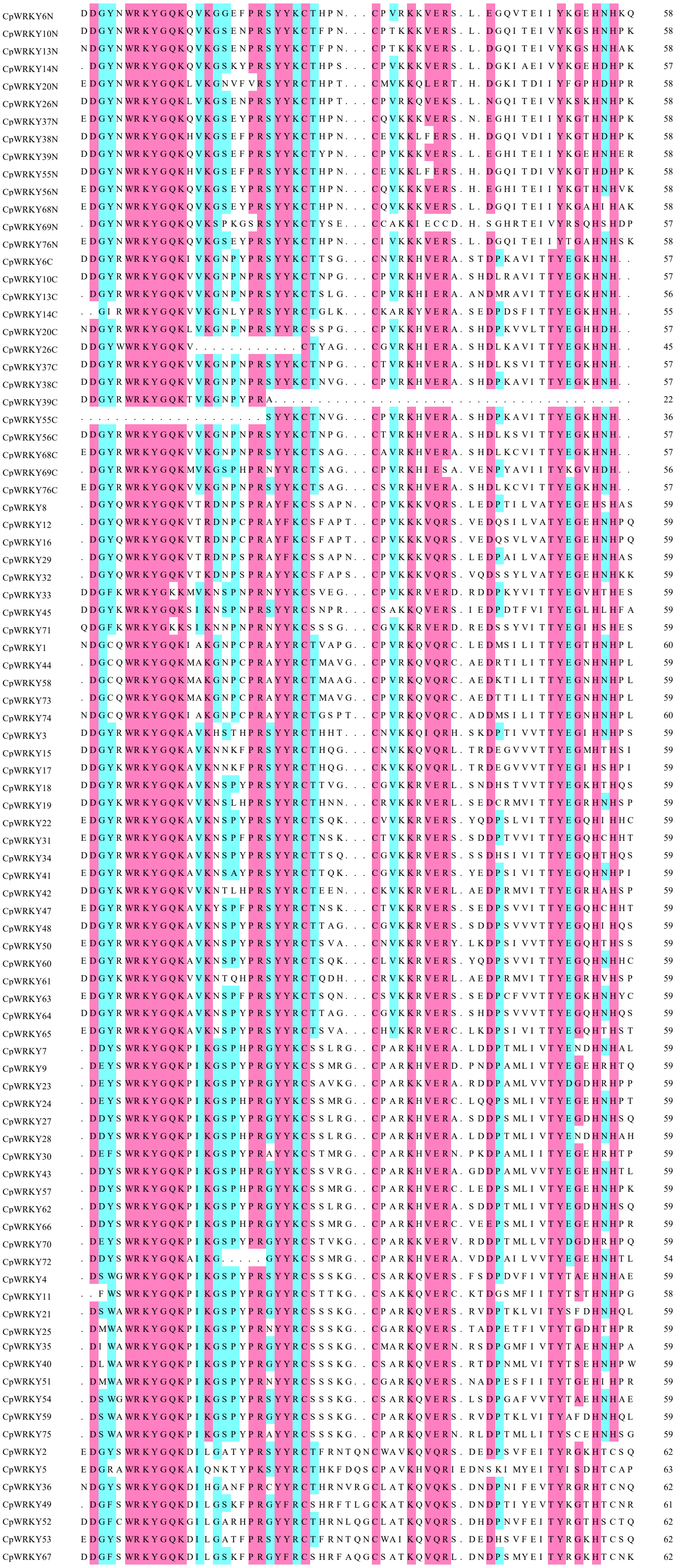

Figure S1 Alignment of 76 CpWRKY WRKY domain sequences.

For Group I WRKY proteins, N-terminal and C-terminal WRKY domains are represented by ‘N’ and ‘C’, respectively. The typical amino acid residues within WRKY domain and zinc-finger motif are in red color.
