# Supplementary material for: Genome-Wide Identification of the WRKY Gene Family and Functional Characterization of CpWRKY5 in Cucurbita pepo
Source: Int J Mol Sci. 2024 Apr 10;25(8):4177. doi: 10.3390/ijms25084177 (PMC11049939; doi:10.3390/ijms25084177)
Supplement: Supplementary file 1 [file ijms-25-04177-s001.zip › Figure S2.pdf]

Motif 1

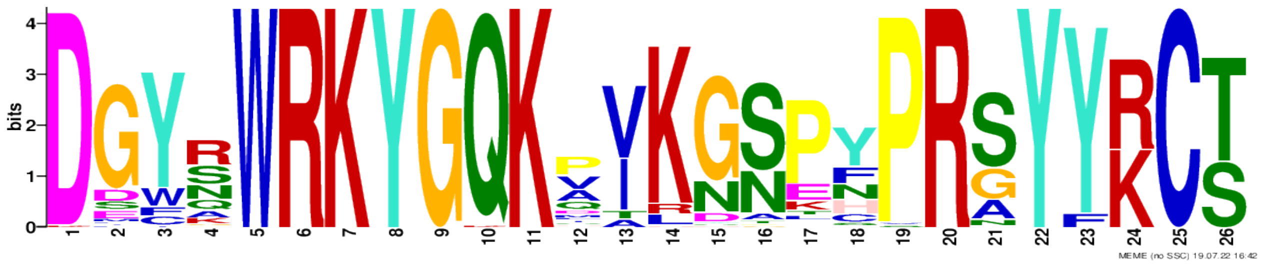

Motif 2

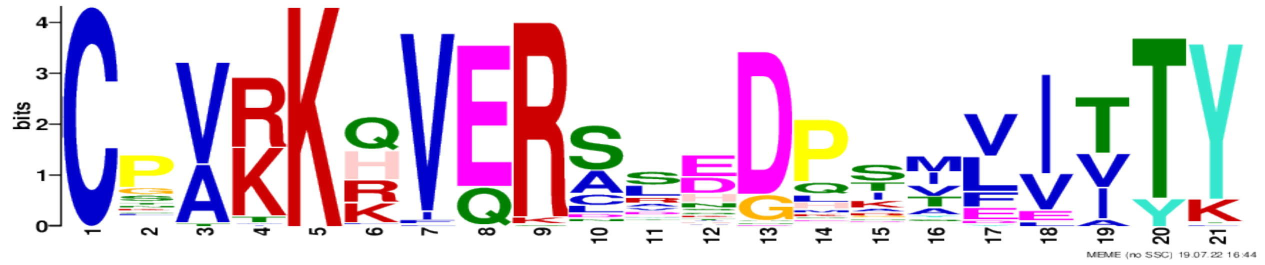

Motif 3

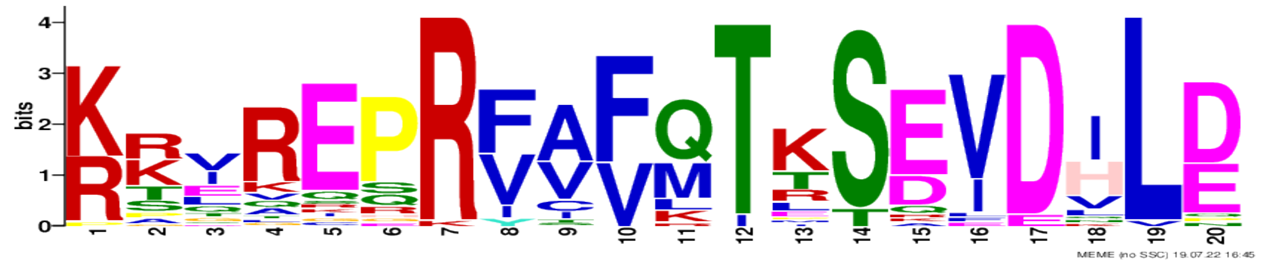

Motif 4

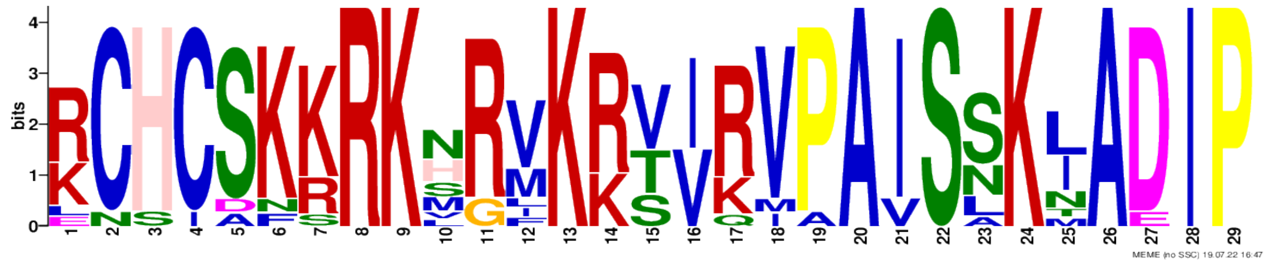

Motif 5

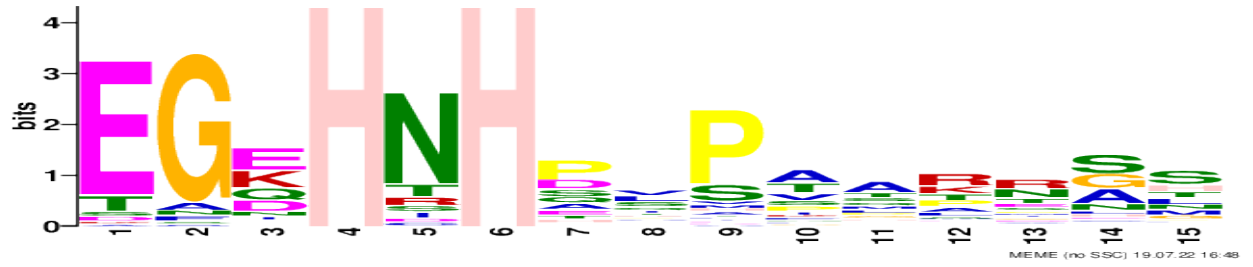

Motif 6

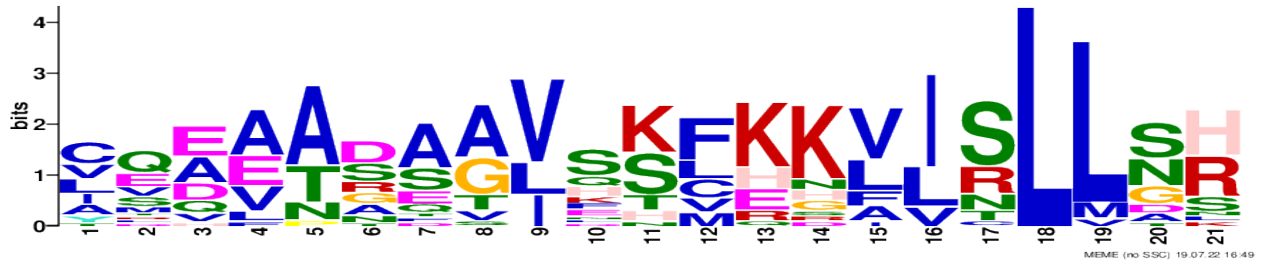

Motif 7

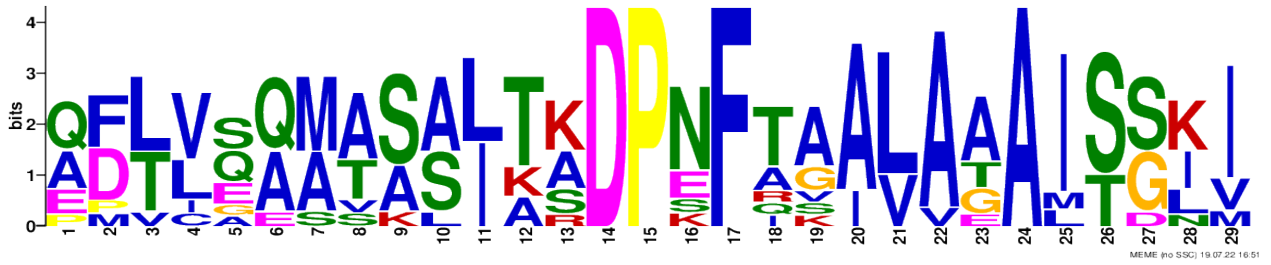

Motif 8

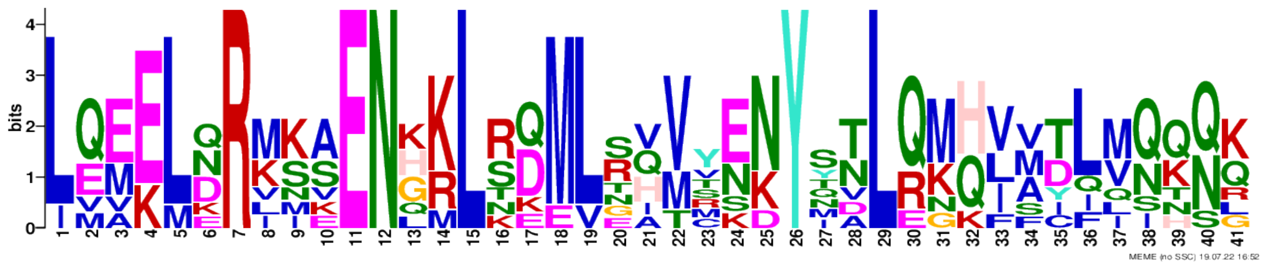

Motif 9

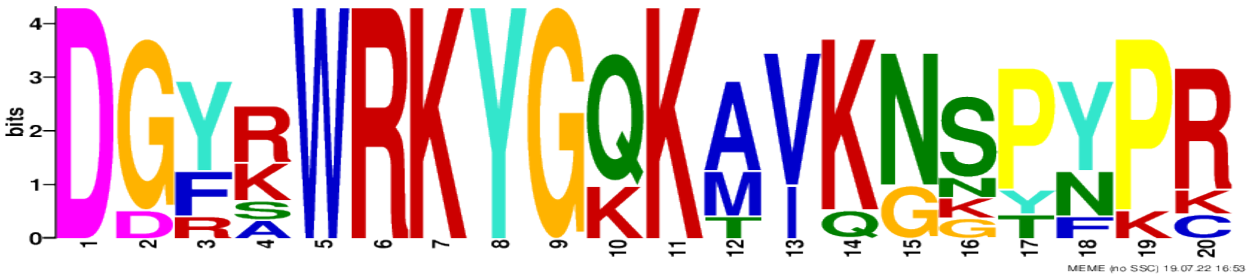

Motif 10

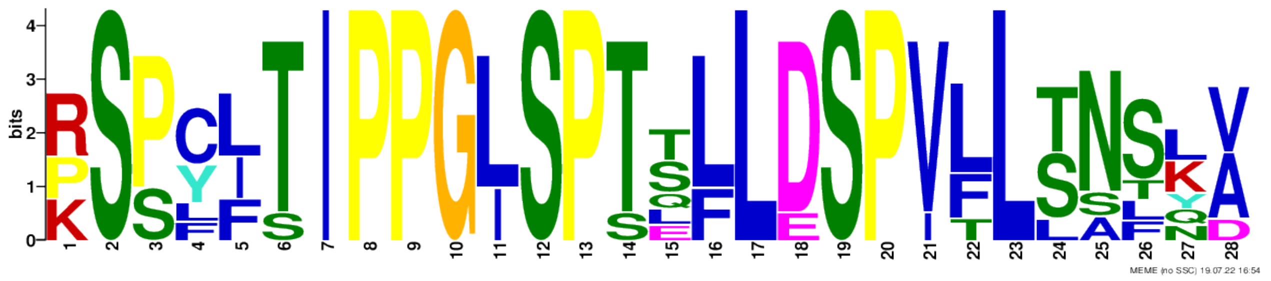

Figure S2 Ten types of conserved motifs in *Cucurbita pepo*.
