# Supplementary material for: Genome-Wide Identification of the WRKY Gene Family and Functional Characterization of CpWRKY5 in Cucurbita pepo
Source: Int J Mol Sci. 2024 Apr 10;25(8):4177. doi: 10.3390/ijms25084177 (PMC11049939; doi:10.3390/ijms25084177)
Supplement: Supplementary file 1 [file ijms-25-04177-s001.zip › Figure S4.pdf]

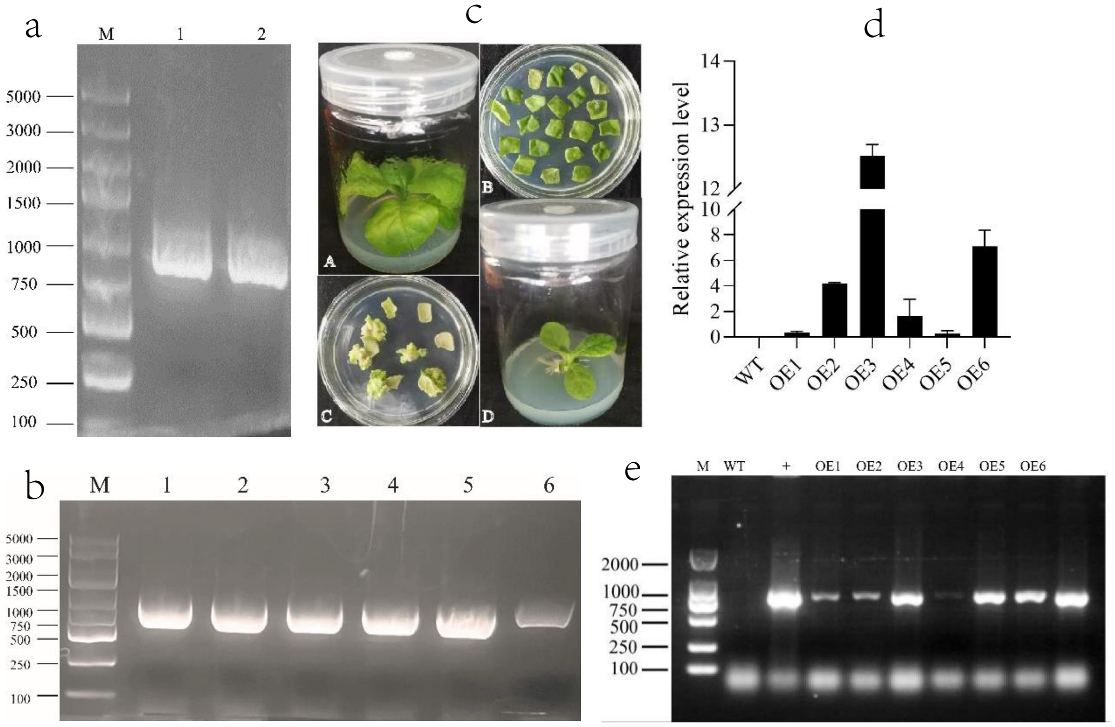

Figure S4 Construction of transgenic tobacco and identification of positive plants

a: *CpWRKY5* gene CDS cloning, b: PCR detection of pCAMBIA2300-CpWRKY5-GFP, c: *Agrobacterium*-mediated SR tobacco genetic transformation (A: Cultivation of sterile tobacco, B: Overexpression of *CpWRKY5* was co-cultured with tobacco, C: Induced differentiation of tobacco overexpressing *CpWRKY5*, D: Rooting culture of tobacco overexpressing *CpWRKY5*), d: qRT-PCR detection of *CpWRKY5* overexpression tobacco, e: *CpWRKY5* overexpression tobacco genomic DNA detection (WT: Wild type SR tobacco, +: pCAMBIA2300 – CpWRKY5 positive plasmid , OE1-OE6: *CpWRKY5* overexpression tobacco)
