# Supplementary figures and images for: Genome-Wide Identification of the WRKY Gene Family and Functional Characterization of CpWRKY5 in Cucurbita pepo
Source: Int J Mol Sci. 2024 Apr 10;25(8):4177. doi: 10.3390/ijms25084177 (PMC11049939; doi:10.3390/ijms25084177)

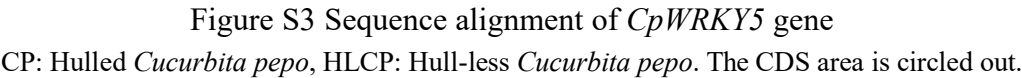

CP: Hulled *Cucurbita pepo*, HLCP: Hull-less *Cucurbita pepo*. The CDS area is circled out.

Supplement: Supplementary file 1 [file ijms-25-04177-s001.zip › Figure S3.pdf]
